# Supplementary material for: Do Multiple Sex/Gender Dimensions Play a Role in the Association of Green Space and Self-Rated Health? Model-Based Recursive Partitioning Results from the KORA INGER Study
Source: Int J Environ Res Public Health. 2023 Mar 23;20(7):5241. doi: 10.3390/ijerph20075241 (PMC10094300; doi:10.3390/ijerph20075241)
Supplement: Supplementary file 1 [file ijerph-20-05241-s001.zip › Supplementary_Material_S1_Results_with_40_Covariates.pdf]

Supplementary Material S1

Containing detailed results of the analyses with 40 sex/gender covariates in the complete case sample (N = 2,534)

Article: " Do multiple sex/gender-dimensions play a role in the association of green space and self-rated health? Model-based recursive partitioning results from the KORA INGER study”

Lisa Dandolo, Klaus Telkmann, Christina Hartig, Sophie Horstmann, Sara Pedron, Lars Schwettmann, Peter Selsam, Alexandra Schneider, Gabriele Bolte on behalf of the INGER Study Group

1. Access to high quality public green spaces (subjectively measured)

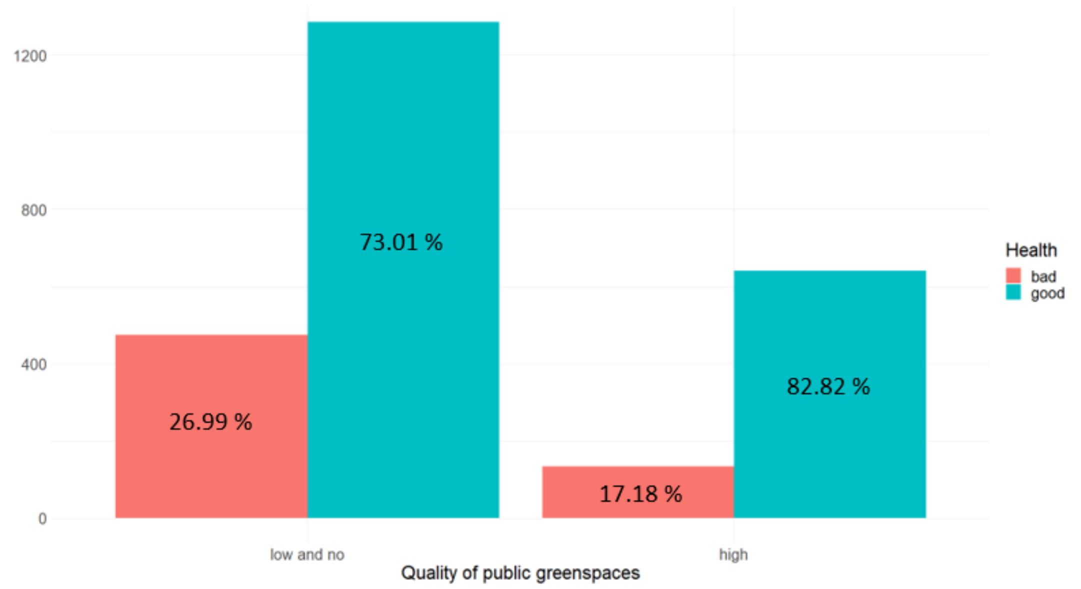

Figure 1: Access to high quality public green space - health association in the complete case sample. Red bars show the number of participants rating their health as bad, and the turquoise bars the number of participants rating their health as good. Bar plots for participants with access to lower quality public green spaces or no access to green spaces are shown on the left side of the figure and bar plots for participants with access to high quality public green spaces on the right side of the figure.

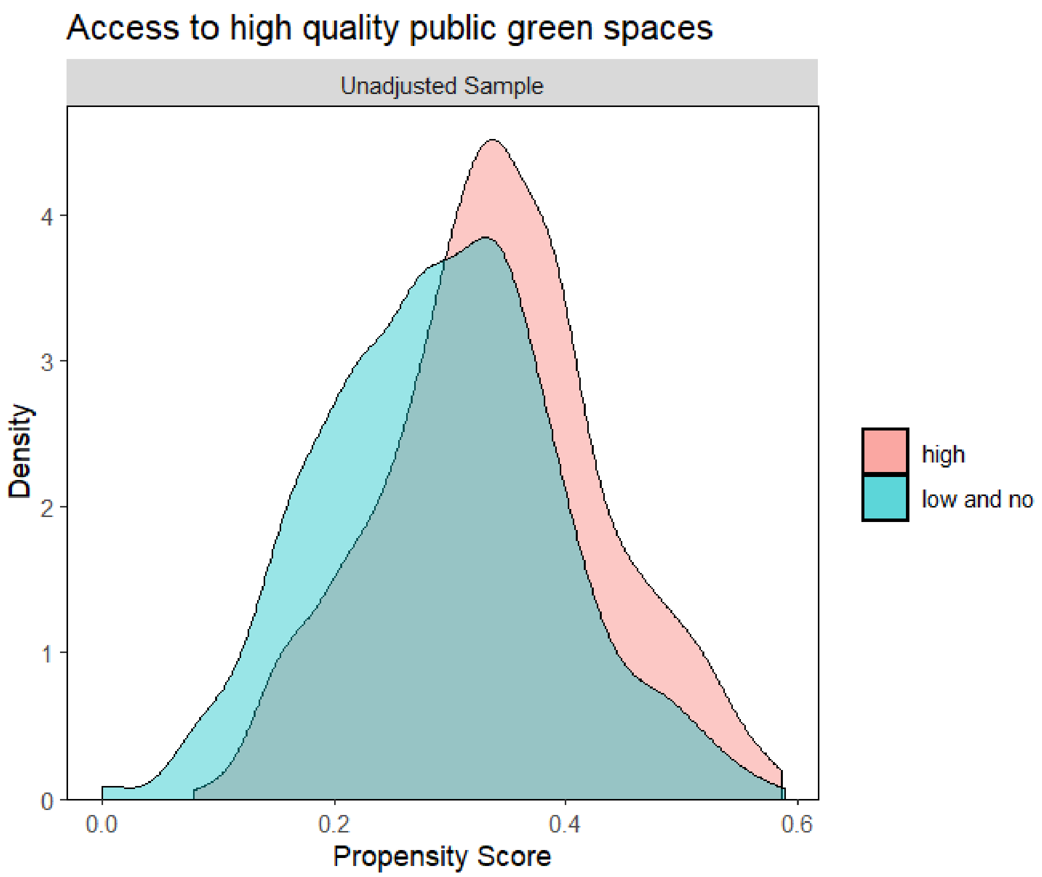

Figure 2: Distribution of propensity scores amongst the exposed and non-exposed.

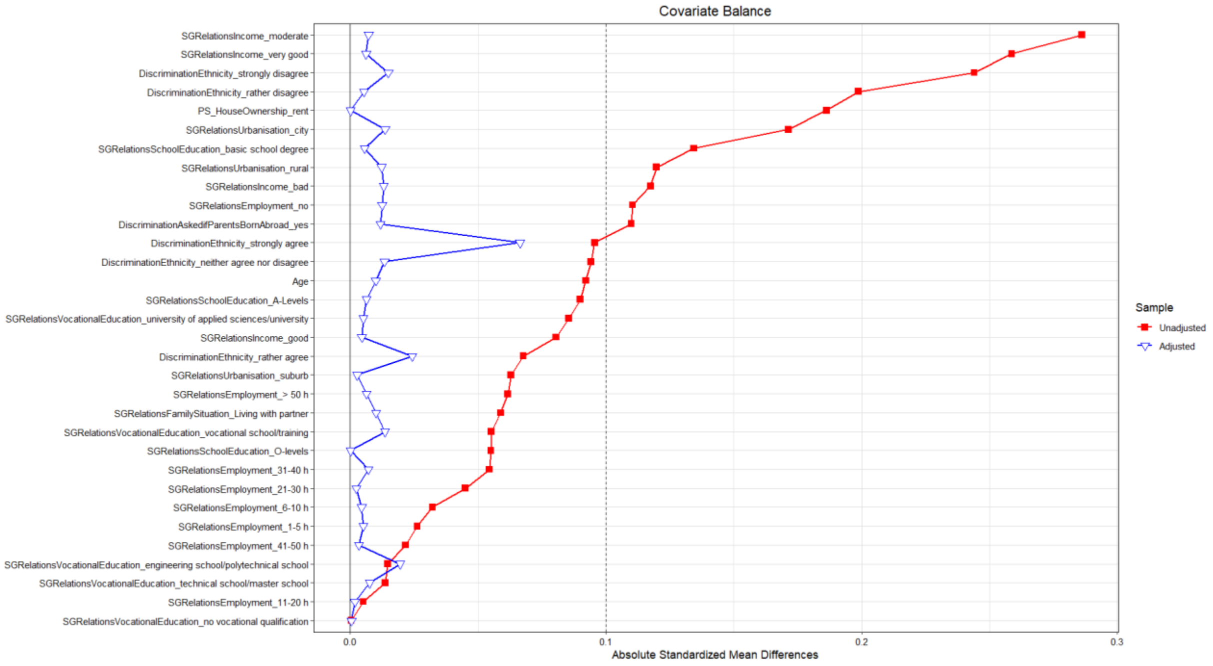

Figure 3: Balance Diagnostics for the "access to high quality public green space" exposure measure showing absolute standardized mean differences before (unadjusted - red line with squares) and after (adjusted – blue line with triangles) weighting. Differences in the adjusted sample were below 0.1 (dotted vertical line) indicating good covariate balance.

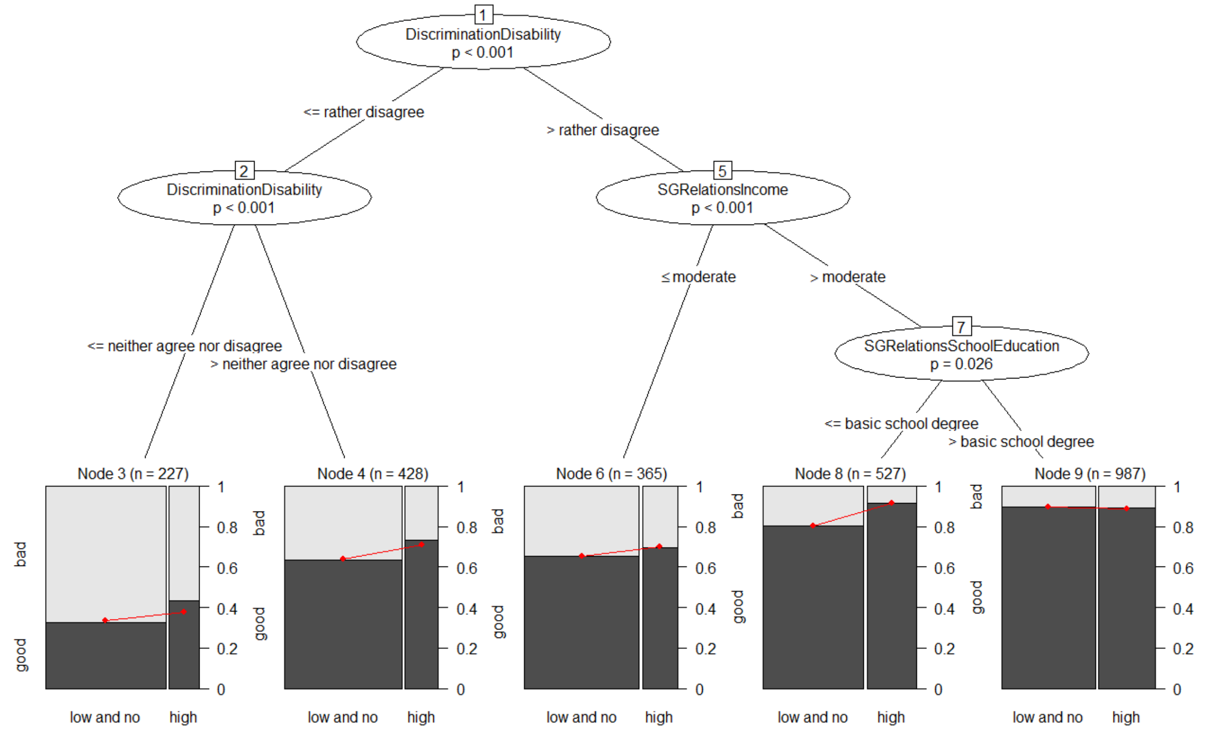

Figure 4: Generalized linear model tree for the "access to high quality public green space" exposure measure. Each terminal node in the bottom row contains a plot of the relationship between exposure and self-rated health. Red lines correspond to parameter estimates (intercept and exposure coefficient) obtained by weighted logistic regressions.

Quality of public greenspaces: Estimates and CIs

	Intercept			Quality of public greenspaces		
Node	Estimate	Lower_CI	Upper_CI,	Estimate	Lower_CI	Upper_CI
1	2.9195320	2.6247825	3.2473803	1.3764760	1.0957787	1.729077
2	1.1432017	0.9594740	1.3621110	1.3576483	0.8954851	2.058336
**3**	**0.5016835**	**0.3654553**	**0.6886926**	**1.2193476**	**0.5979609**	**2.486464**
**4**	**1.7700808**	**1.4123223**	**2.2184637**	**1.3701342**	**0.7860863**	**2.388119**
5	4.6757566	4.0472740	5.4018335	1.2680332	0.9536826	1.685999
**6**	**1.8905738**	**1.4760137**	**2.4215693**	**1.2335088**	**0.7188601**	**2.116607**
7	6.3503187	5.2835865	7.6324192	1.3706788	0.9766184	1.923741
**8**	**4.1037425**	**3.1521269**	**5.3426474**	**2.6556832**	**1.4364658**	**4.909726**
**9**	**8.6346101**	**6.6502961**	**11.2110032**	**0.9114754**	**0.5971494**	**1.391255**

Table 1: Parameter estimates and corresponding upper and lower bounds of the 95% confidence intervals obtained by weighted logistic regressions on exponential scale for each node of the tree depicted in Figure 4 for the "access to high quality public green space" exposure measure. Node 1 is the root node and contains the whole dataset. Terminal nodes are shown in bold.

Quality pf public greenspaces: Risk differences

Node	RD	Lower_CI	Upper_CI	P0	P1
1	0.0558764	0.0231739	0.0886085	0.7448675	0.8007439
2	0.0747522	-0.0107675	0.1571780	0.5334084	0.6081606
**3**	**0.0454667**	**-0.0882837**	**0.1924982**	**0.3340807**	**0.3795474**
**4**	**0.0690507**	**-0.0275039**	**0.1648793**	**0.6389997**	**0.7080504**
5	0.0318673	-0.0001835	0.0625750	0.8238120	0.8556793
**6**	**0.0458356**	**-0.0463655**	**0.1429361**	**0.6540479**	**0.6998835**
7	0.0330009	0.0047525	0.0630635	0.8639515	0.8969523
**8**	**0.1118886**	**0.0605700**	**0.1607682**	**0.8040654**	**0.9159540**
**9**	**-0.0089441**	**-0.0446187**	**0.0237438**	**0.8962075**	**0.8872634**

Table 2: Estimates of risk differences (RD) between exposed and non-exposed. Confidence intervals are based on 2000 bootstrap samples. P0 and P1 are estimates of the probabilites of good self-rated health among the exposed and non-exposed respectively, i.e. $P0 = \mathbb{P}[Y \mid E = 0]$ *and* $P1 = \mathbb{P}[Y \mid E = 1]$*. Node 1 is the root node and contains the whole dataset. Terminal nodes are depicted in bold.*

2. Greenness in the residential environment (subjectively measured)

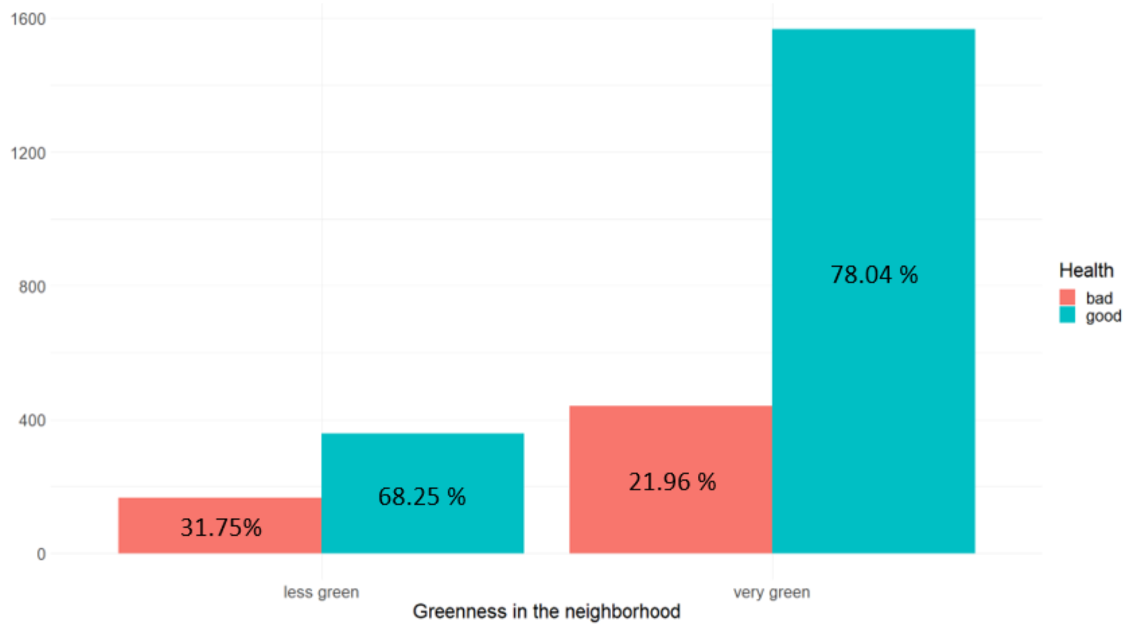

Figure 5: Greenness in the residential environment - health association in the complete case sample. Red bars show the number of participants rating their health as bad, and the turquoise bars the number of participants rating their health as good. Bar plots for participants with a less green self-rated residential environment are shown on the left side of the figure and bar plots for participants with a very green self-rated residential environment on the right side of the figure.

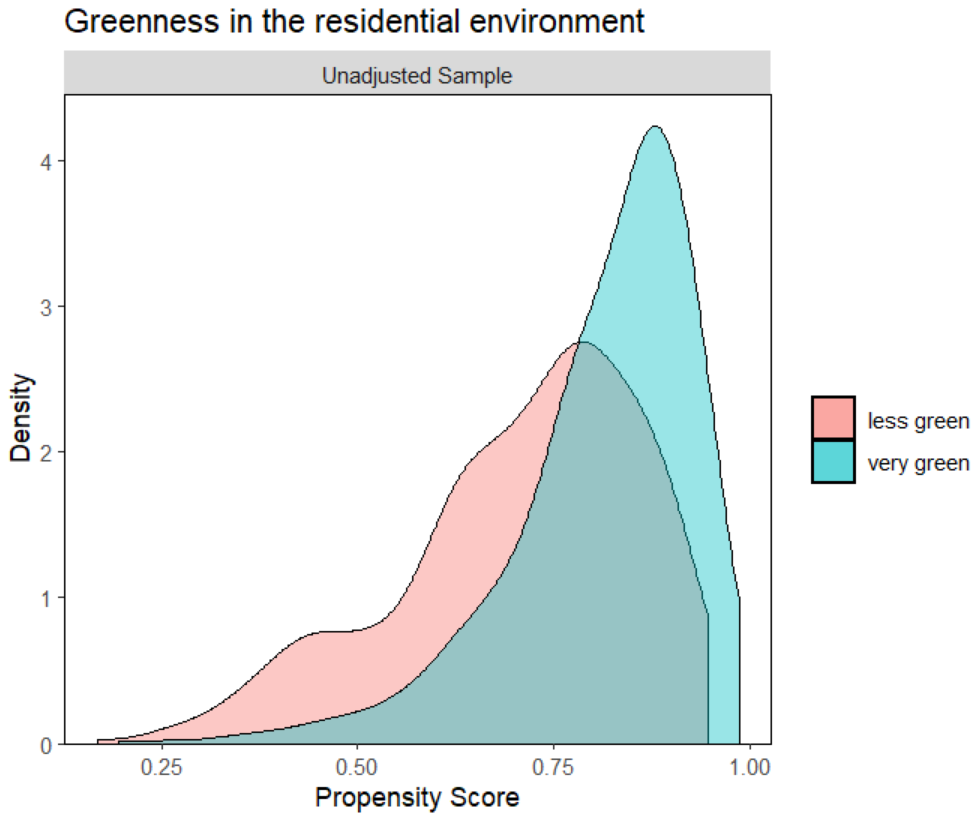

Figure 6: Distribution of propensity scores amongst the exposed and non-exposed.

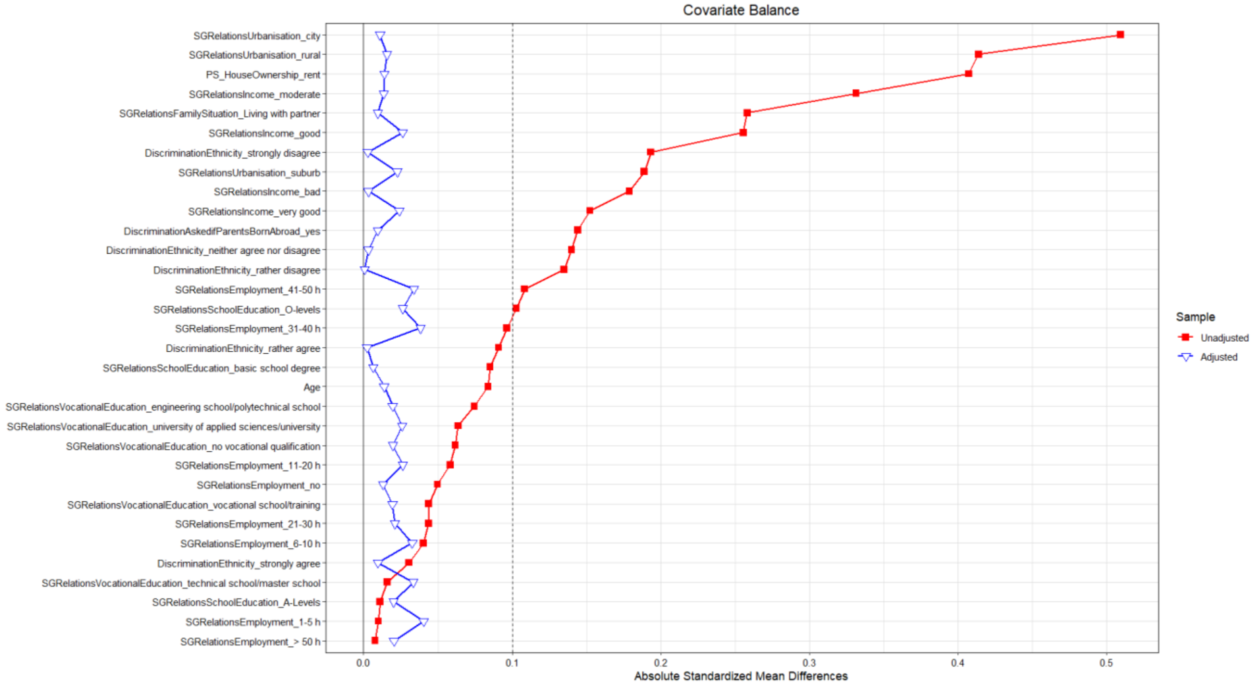

Figure 7: Balance Diagnostics for the "greenness in the residential environment" exposure measure showing absolute standardized mean differences before (unadjusted - red line with squares) and after (adjusted – blue line with triangles) weighting. Differences in the adjusted sample were below 0.1 (dotted vertical line) indicating good covariate balance.

.

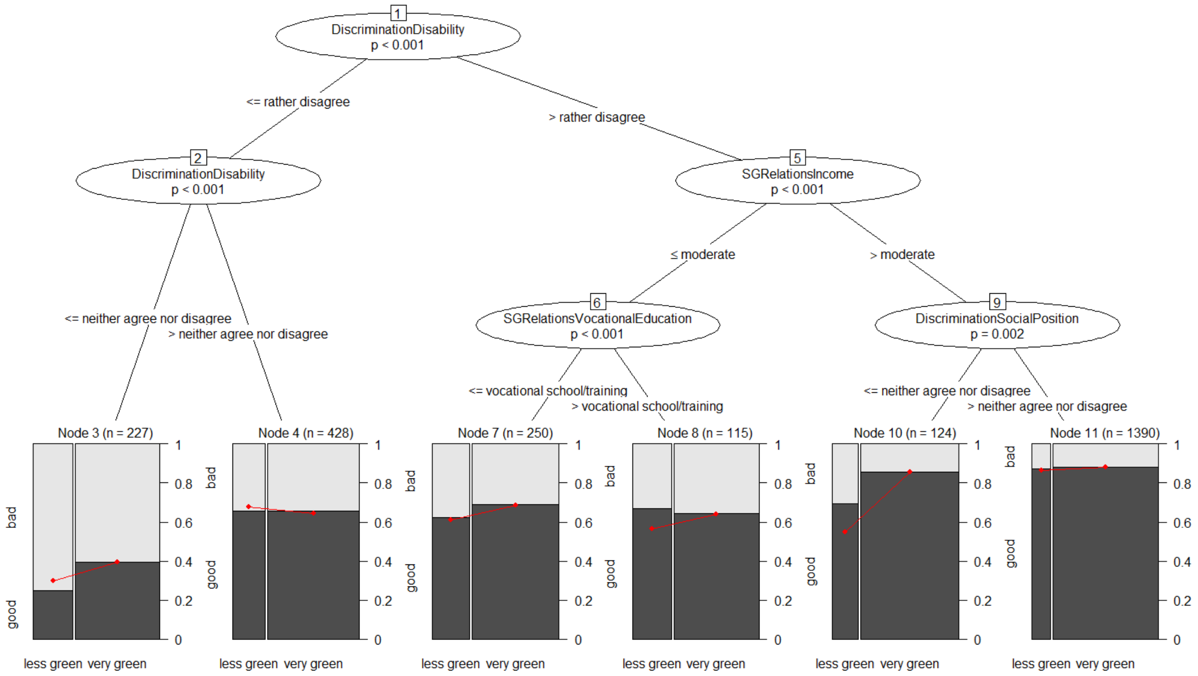

Figure 8: Generalized linear model tree for exposure „greenness in the residential environment". Each terminal node in the bottom row contains a plot of the relationship between exposure and self-rated health. Red lines correspond to parameter estimates (intercept and exposure coefficient) obtained by weighted logistic regressions.

Greenness in the residential environment: Estimates and CIs

	Intercept			Greenness		
Node	Estimate	Lower_CI	Upper_CI,	Estimate	Lower_CI	Upper_CI
1	2.3843718	1.8949167	3.0002526	1.3932988	1.0804137	1.796795
2	1.1656003	0.8218014	1.6532266	1.1005547	0.7388610	1.639308
**3**	**0.4238731**	**0.2129298**	**0.8437918**	**1.5353510**	**0.7094882**	**3.322539**
**4**	**2.0827191**	**1.2930572**	**3.3546225**	**0.8656764**	**0.5072198**	**1.477457**
5	3.4849685	2.5135699	4.8317755	1.4812835	1.0377560	2.114371
6	1.4741375	0.9380993	2.3164727	1.3877774	0.8192256	2.350910
**7**	**1.5696479**	**0.9215132**	**2.6736402**	**1.3955222**	**0.7452397**	**2.613229**
**8**	**1.2996415**	**0.5421872**	**3.1152857**	**1.3611999**	**0.5022664**	**3.689009**
9	4.6807726	2.9905993	7.3261675	1.5279514	0.9466499	2.466208
**10**	**1.2047175**	**0.4005909**	**3.6230086**	**4.9021698**	**1.4156318**	**16.975649**
**11**	**6.4180624**	**3.9126035**	**10.5279068**	**1.1333433**	**0.6700582**	**1.916948**

Table 3: Parameter estimates and corresponding upper and lower bounds of the 95% confidence intervals obtained by weighted logistic regressions on exponential scale for each node of the tree depicted in Figure 8 for the "greenness in the residential environment" exposure measure. Node 1 is the root node and contains the whole dataset. Terminal nodes are shown in bold.

Greenness in the residential environment: Risk differences

Node	RD	Lower_CI	Upper_CI	P0	P1
1	0.0641091	0.0211269	0.1092154	0.7045242	0.7686333
2	0.0237085	-0.0578538	0.1117356	0.5382343	0.5619428
**3**	**0.0965407**	**-0.0433180**	**0.2228644**	**0.2976902**	**0.3942309**
**4**	**-0.0323767**	**-0.1234984**	**0.0651710**	**0.6756111**	**0.6432344**
5	0.0606880	0.0124733	0.1116130	0.7770330	0.8377210
6	0.0758576	-0.0294541	0.1737560	0.5958187	0.6716763
**7**	**0.0757258**	**-0.0422933**	**0.2015755**	**0.6108416**	**0.6865674**
**8**	**0.0737186**	**-0.1227775**	**0.2544742**	**0.5651496**	**0.6388682**
9	0.0533630	-0.0003121	0.1121517	0.8239676	0.8773306
**10**	**0.3087656**	**0.0780073**	**0.5115635**	**0.5464271**	**0.8551927**
**11**	**0.0139436**	**-0.0339684**	**0.0674156**	**0.8651939**	**0.8791375**

Table 4: Estimates of risk differences (RD) between exposed and non-exposed. Confidence intervals are based on 2000 bootstrap samples. P0 and P1 are estimates of the probabilites of good self-rated health among the exposed and non-exposed respectively, i.e. $P0 = \mathbb{P}[Y \mid E = 0]$ *and* $P1 = \mathbb{P}[Y \mid E = 1]$*. Node 1 is the root node and contains the whole dataset. Terminal nodes are depicted in bold.*

3. Greenness within a 300 m buffer around the residential address (objectively measured)

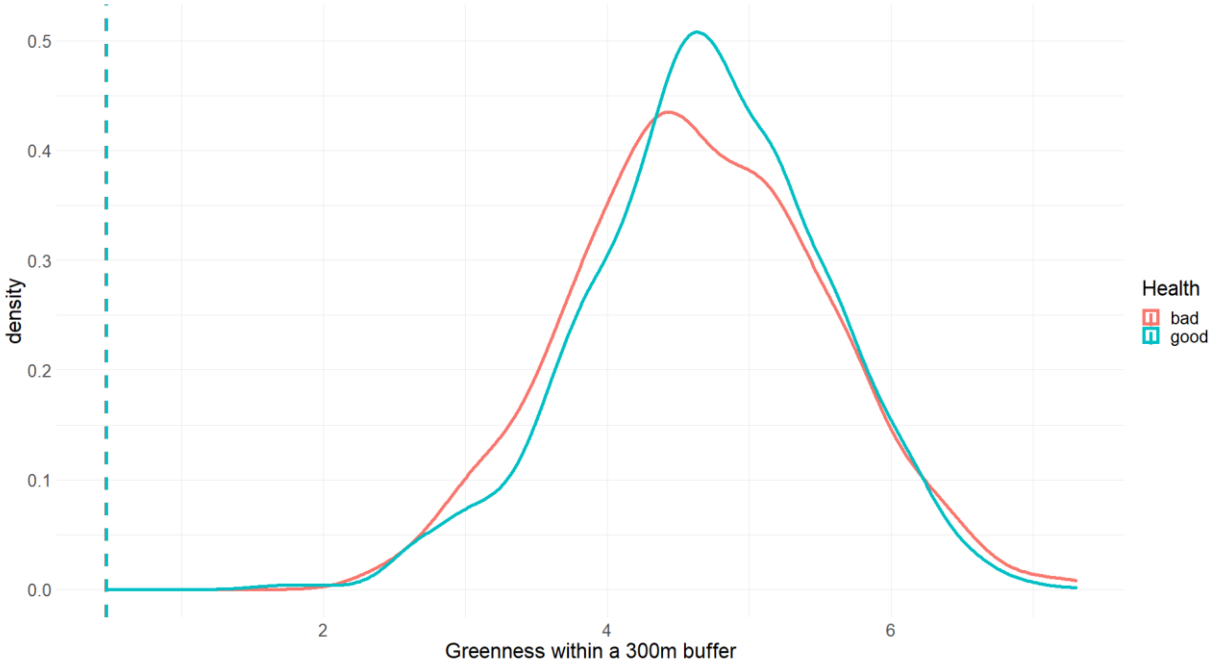

Figure 9: Greenness within a 300 m buffer around the residential address - health association in the complete case sample. The red density plot shows the distribution of the NDVI data for participants rating their health as bad, and the turquoise density plot shows the distribution of the NDVI data for participants rating their health as good.

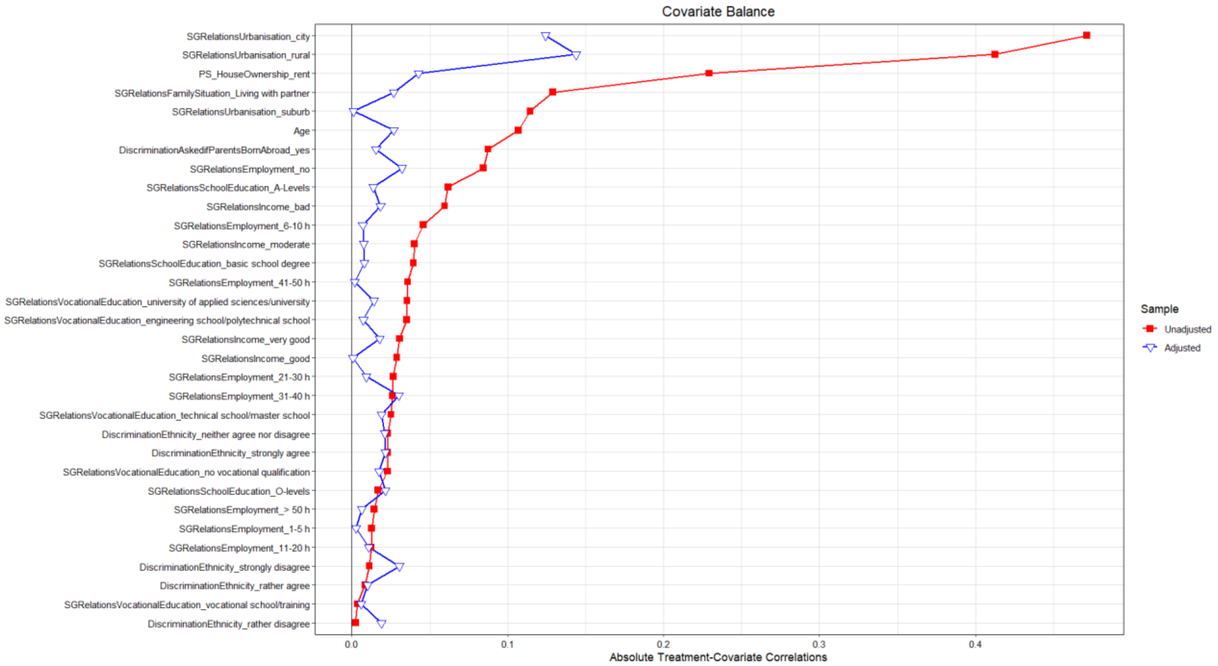

Figure 10: Balance Diagnostics for the "greenness within a 300 m buffer around the residential address" exposure measure showing absolute treatment-covariate correlations before (unadjusted - red line with squares) and after (adjusted – blue line with triangles) weighting.

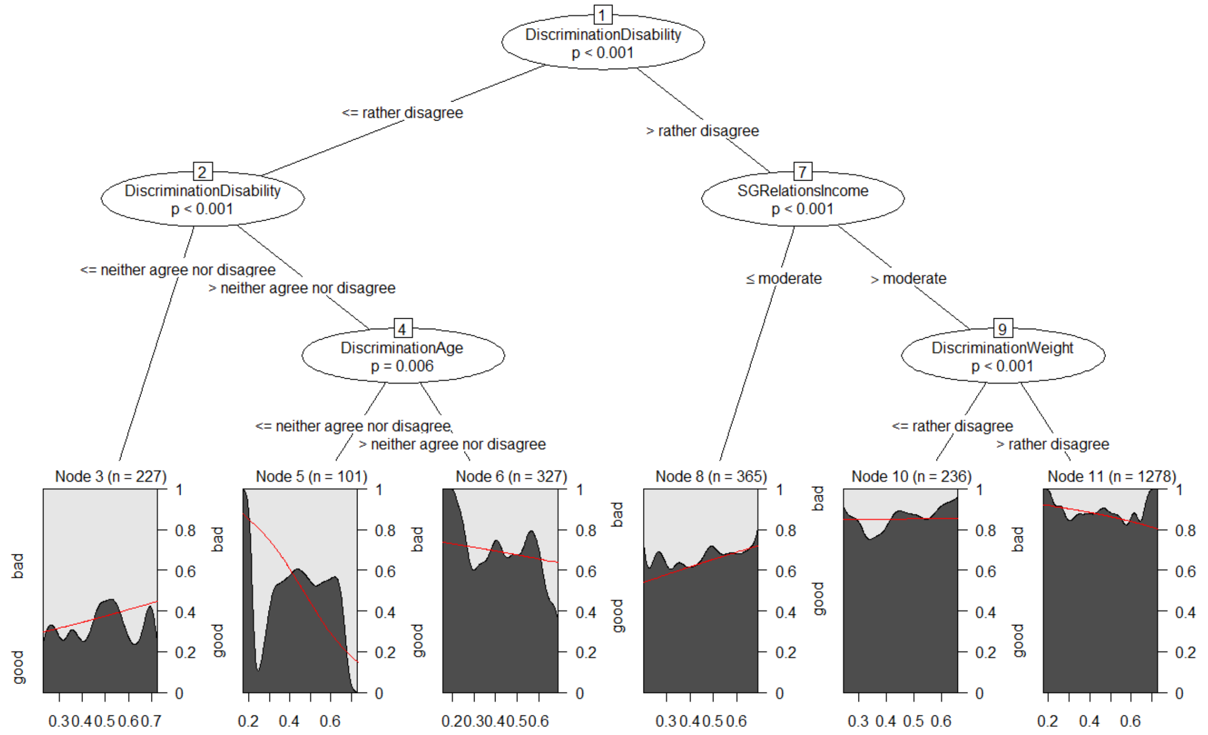

Figure 11: Generalized linear model tree for exposure „greenness within a 300 m buffer around the residential address ". Each terminal node in the bottom row contains a conditional density plot of the relationship between exposure and self-rated health. Red lines correspond to probability curves obtained by weighted logistic regressions.

NDVI 300: Estimates and CIs

	Intercept			NDVI_300		
Node	Estimate	Lower_CI	Upper_CI,	Estimate	Lower_CI	Upper_CI
1	3.3872292	1.6840494	6.812936	0.9739733	0.8386715	1.1311030
2	1.7211227	0.5610571	5.279789	0.9236201	0.7262401	1.1746446
**3**	**0.3091426**	**0.0386274**	**2.474132**	**1.1418752**	**0.7351125**	**1.7737136**
4	5.9322483	1.3785276	25.528375	0.7706784	0.5626729	1.0555782
**5**	**21.9807122**	**1.2694748**	**380.591811**	**0.5145318**	**0.2793564**	**0.9476891**
**6**	**3.2413504**	**0.5501930**	**19.095760**	**0.9152251**	**0.6247429**	**1.3407709**
7	4.7922329	1.8790091	12.222132	0.9914560	0.8112784	1.2116495
**8**	**0.8516234**	**0.1498369**	**4.840345**	**1.1722841**	**0.7992897**	**1.7193391**
9	13.1794713	4.4036627	39.444089	0.8597681	0.6816173	1.0844814
**10**	**5.3486762**	**0.4257056**	**67.202165**	**1.0151464**	**0.5849789**	**1.7616400**
**11**	**16.1958808**	**4.7269443**	**55.491781**	**0.8276505**	**0.6384704**	**1.0728851**

Table 5: Parameter estimates and corresponding upper and lower bounds of the 95% confidence intervals obtained by weighted logistic regressions on exponential scale for each node of the tree depicted in Figure 11 for the "greenness within a 300 m buffer around the residential address" exposure measure. Node 1 is the root node and contains the whole dataset. Terminal nodes are shown in bold.

4. Greenness within a 1000 m buffer around the residential address (objectively measured)

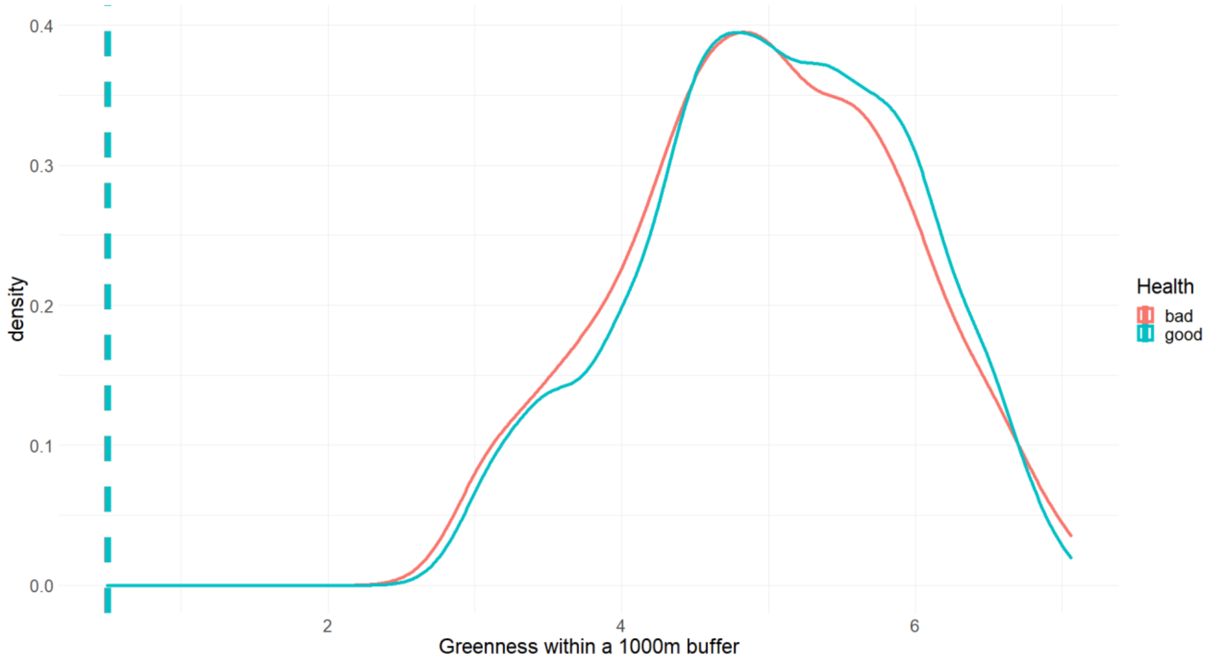

Figure 12: Greenness within a 1000 m buffer around the residential address - health association in the complete case sample. The red density plot shows the distribution of the NDVI data for participants rating their health as bad, and the turquoise density plot shows the distribution of the NDVI data for participants rating their health as good.

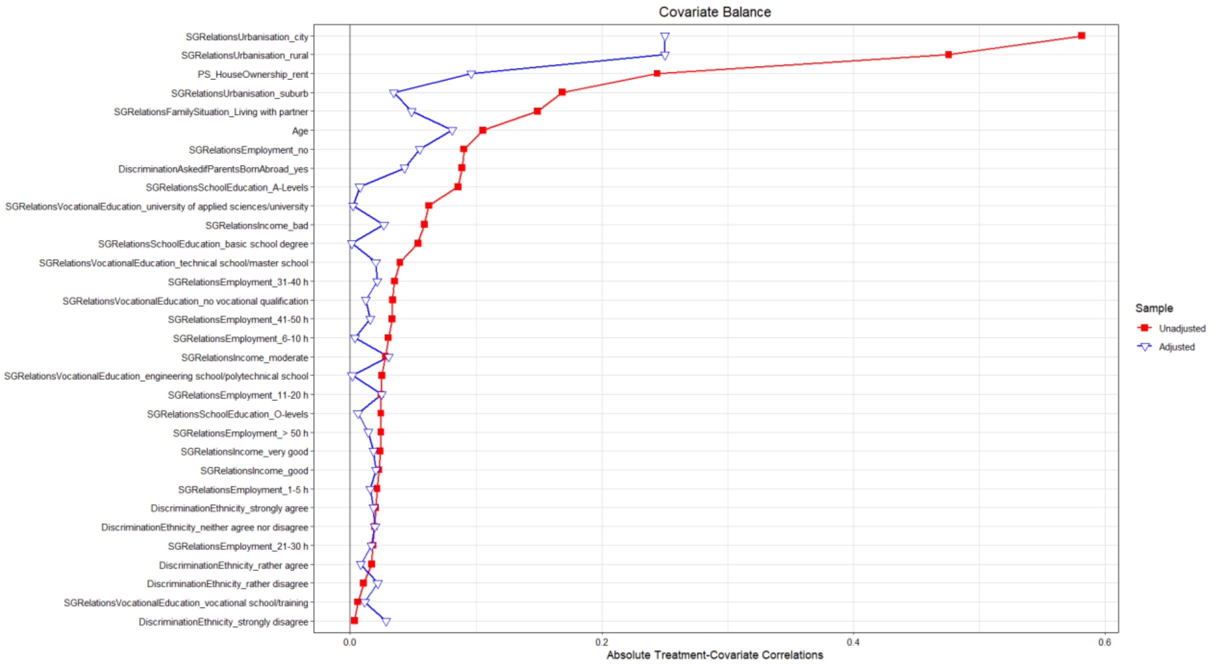

Figure 13: Balance Diagnostics for the "greenness within a 1000 m buffer around the residential address" exposure measure showing absolute treatment-covariate correlations before (unadjusted - red line with squares) and after (adjusted – blue line with triangles) weighting.

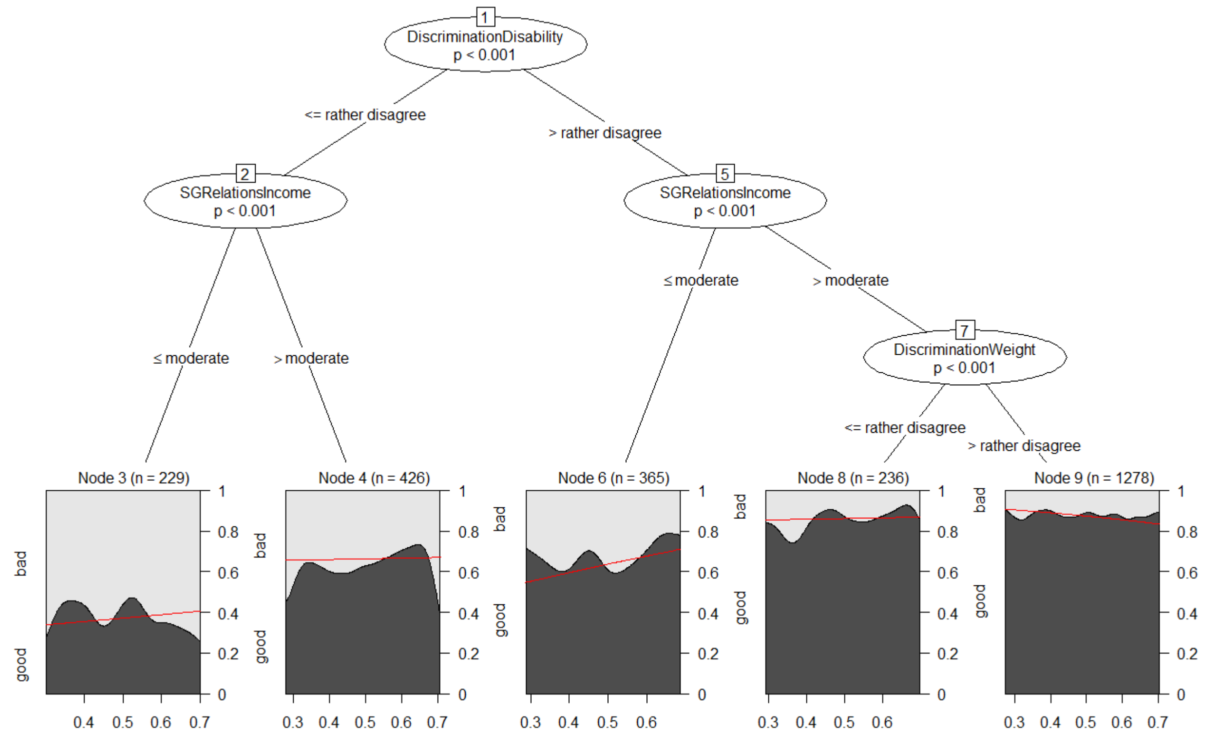

Figure 14: Generalized linear model tree for exposure „ greenness within a 1000 m buffer around the residential address ". Each terminal node in the bottom row contains a conditional density plot of the relationship between exposure and self-rated health. Red lines correspond to probability curves obtained by weighted logistic regressions.

NDVI 1000: Estimates and CIs

	Intercept			NDVI_1000		
Node	Estimate	Lower_CI	Upper_CI,	Estimate	Lower_CI	Upper_CI
1	2.8083577	1.4151385	5.573216	1.0227262	0.8936151	1.170492
2	1.0608097	0.3113309	3.614538	1.0431023	0.8200245	1.326866
**3**	**0.4123481**	**0.0665135**	**2.556338**	**1.0748123**	**0.7527218**	**1.534726**
**4**	**1.8216477**	**0.3954504**	**8.391446**	**1.0156166**	**0.7468252**	**1.381149**
5	4.6082624	1.9625403	10.820711	1.0066939	0.8507321	1.191248
**6**	**0.7261885**	**0.1459046**	**3.614346**	**1.1928420**	**0.8703880**	**1.634756**
7	12.7653815	4.8572327	33.548931	0.8809826	0.7264504	1.068387
**8**	**5.3895287**	**0.7515388**	**38.650061**	**1.0285699**	**0.6832138**	**1.548499**
**9**	**14.9686912**	**4.9946532**	**44.860315**	**0.8561474**	**0.6885814**	**1.064491**

Table 6: Parameter estimates and corresponding upper and lower bounds of the 95% confidence intervals obtained by weighted logistic regressions on exponential scale for each node of the tree depicted in Figure 14 for the "greenness within a 1000 m buffer around the residential address" exposure measure. Node 1 is the root node and contains the whole dataset. Terminal nodes are shown in bold.

Exposure	Mean	SD	Min	Max
Greenness in the residential environment	1.0003470	0.3673546	0.2495532	4.068598
Access to high quality public greenspaces	0.9982672	0.2403831	0.5209226	3.891137
NDVI 300m	0.9398047	0.5692543	0.1065816	4.051978
NDVI 1000m	0.8757361	0.6042800	0.1396599	4.581310

Table 7: Descriptive statistics of the stabilized inverse probability weights. A mean far from 1 and very extreme values indicate violations of the positivity assumption (see Cole and Hernán 2008).
