# Supplementary material for: Do Multiple Sex/Gender Dimensions Play a Role in the Association of Green Space and Self-Rated Health? Model-Based Recursive Partitioning Results from the KORA INGER Study
Source: Int J Environ Res Public Health. 2023 Mar 23;20(7):5241. doi: 10.3390/ijerph20075241 (PMC10094300; doi:10.3390/ijerph20075241)
Supplement: Supplementary file 1 [file ijerph-20-05241-s001.zip › Supplementary_Material_S3_Containing_Supplementary_Fig1-2_Tab1-3.pdf]

Supplementary Material S3

Containing Supplementary Figures S1-S2 and Supplementary Tables S1-S3

Article: " Do multiple sex/gender-dimensions play a role in the association of green space and self-rated health? Model-based recursive partitioning results from the KORA INGER study"

Lisa Dandolo, Klaus Telkmann, Christina Hartig, Sophie Horstmann, Sara Pedron, Lars Schwettmann, Peter Selsam, Alexandra Schneider, Gabriele Bolte on behalf of the INGER Study Group

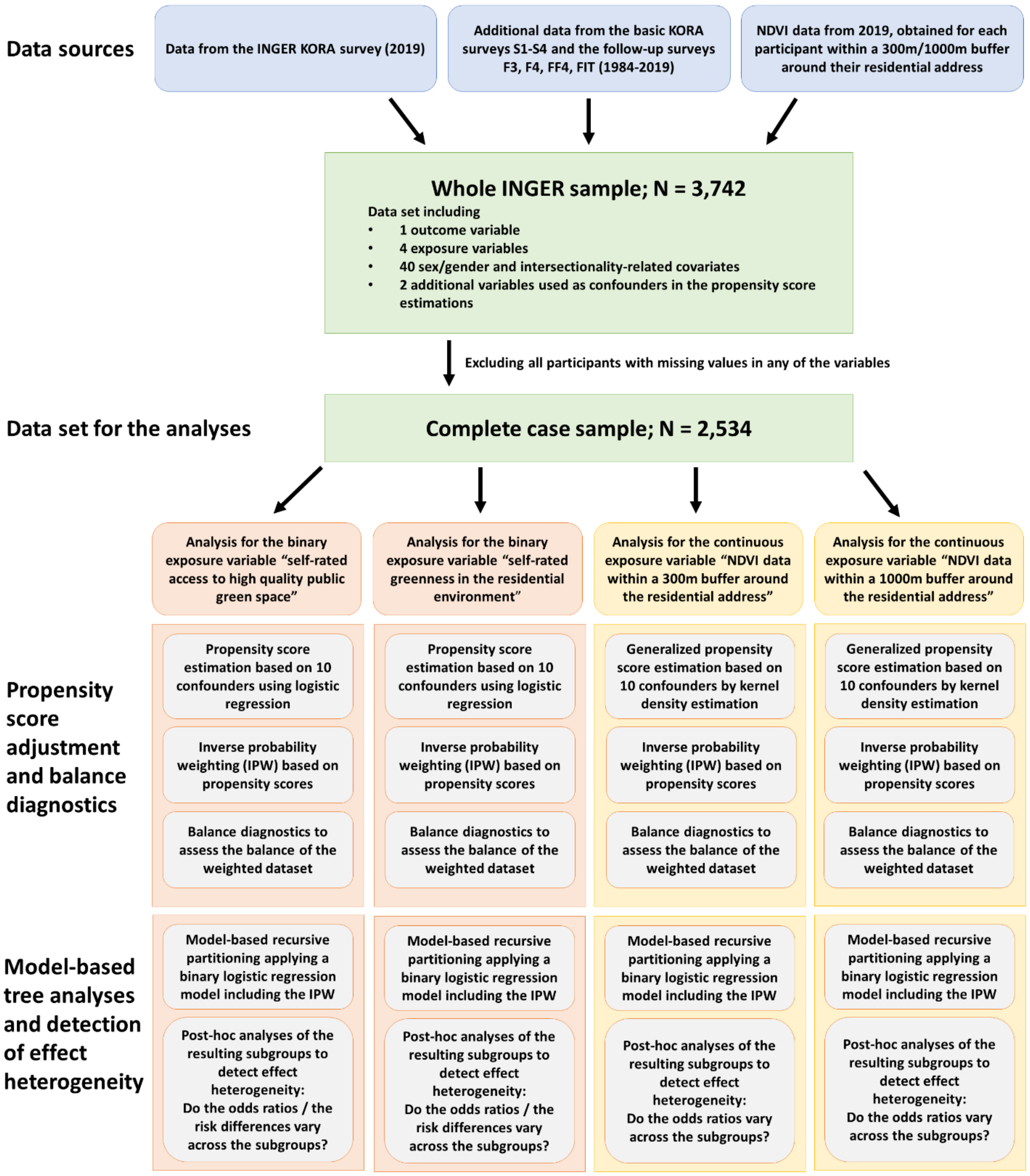

Supplementary Figure S1: *A research structure diagram to visually summarise the research process of our main analyses with 40 sex/gender and intersectionality-related covariates*

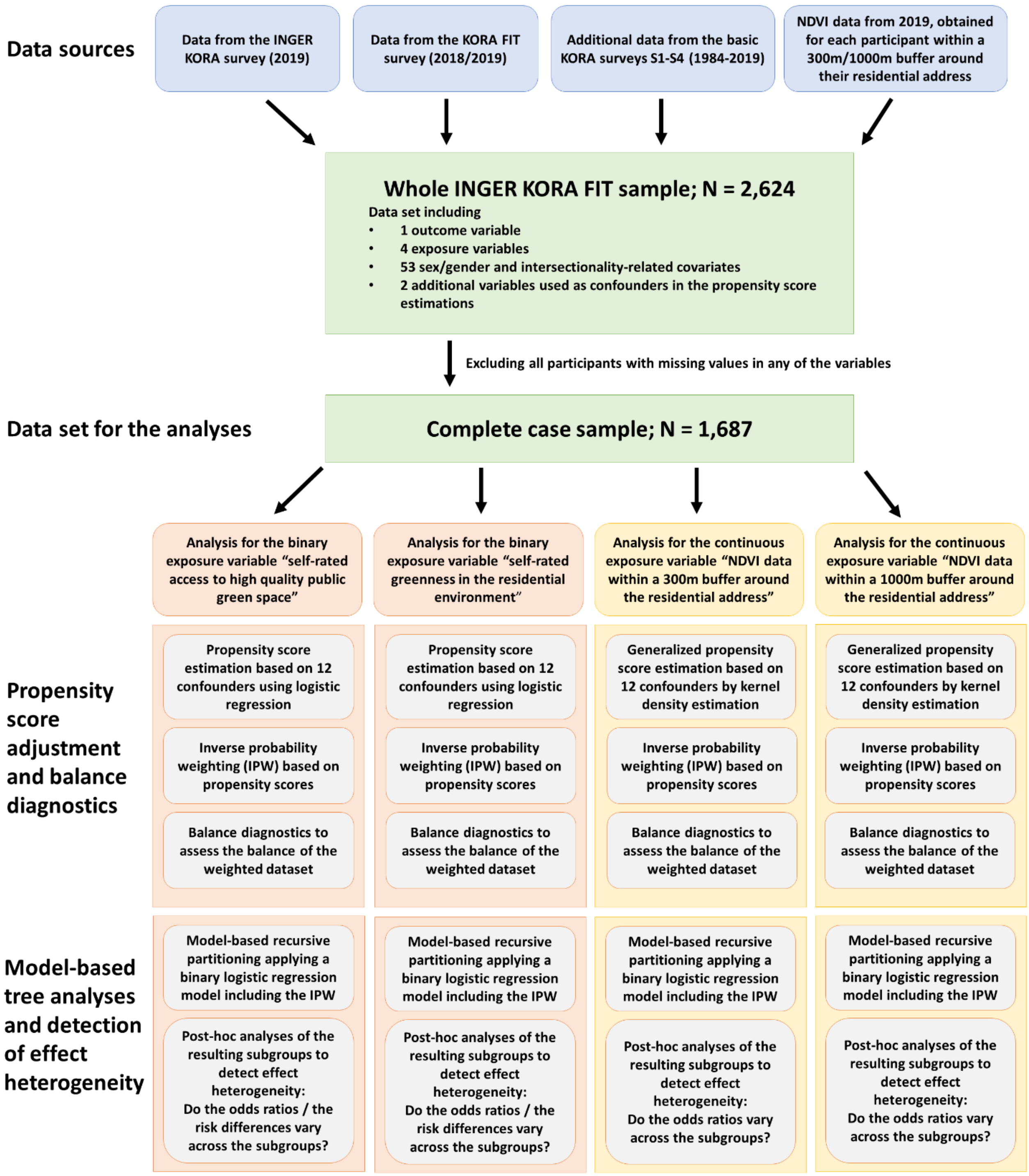

Supplementary Figure S2: *A research structure diagram to visually summarise the research process of our additional analyses for the INGER KORA FIT subsample with 53 sex/gender and intersectionality-related covariates*

Supplementary Table S1: *Distribution of the 40 sex/gender and intersectionality-related covariates in the complete case sample*

**Covariate name and question in INGER KORA survey**[1]	**Response categories and distribution in the complete case sample; *N* = 2,543 (100 %)**	
**<u>Individual sex/gender self-concept</u>**		
**Sex assigned at birth**		
**SexAtBirth**<br>What sex were you assigned at birth?	1,339 (52.8)<br>1,195 (47.2)	= female<br>= male
**Current sex/gender identity**		
**SGIdentity**<br>What is your current sex/gender identity?<br>*(Multiple responses possible)*	1,334 (52.6)<br>1,184 (46.7)<br>2 (0.1)<br>0 (0.0)<br>8 (0.3)<br>2 (0.1)<br>2 (0.1)<br>2 (0.1)	= female<br>= male<br>= trans, trans man, trans woman<br>= an identity not mentioned here<br>= I do not want to classify as any sex/gender category<br>= female AND I do not want to classify as any sex/gender category<br>= male AND an identity not mentioned here<br>= male AND I do not want to classify as any sex/gender category
**Internalized sex/gender roles**		
**SGRolesFemMascuFeeling**<br>I mostly perceive myself as …	299 (11.8)<br>646 (25.5)<br>213 (8.4)<br>293 (11.6)<br>118 (4.7)<br>664 (26.2)<br>301 (11.9)	= very masculine<br>= mainly masculine<br>= a little masculine<br>= just as feminine as masculine<br>= a little feminine<br>= mainly feminine<br>= very feminine
**SGRolesFemMascuWish**<br>Ideally, I would like to be ...	362 (14.3)<br>636 (25.1)<br>132 (5.2)<br>288 (11.4)<br>112 (4.4)<br>622 (24.5)<br>382 (15.1)	= very masculine<br>= mainly masculine<br>= a little masculine<br>= just as feminine as masculine<br>= a little feminine<br>= mainly feminine<br>= very feminine
**SGRolesFemMascuChange**<br>Has your assessment of what is feminine or masculine changed in recent years?	264 (10.4)<br>2,270 (89.6)	= yes<br>= no
**SGRolesBothIncomeContribute**<br>Both the men and women should contribute to the household income.	1,163 (45.9)<br>707 (27.9)<br>575 (22.7)<br>72 (2.8)<br>17 (0.7)	= strongly agree<br>= rather agree<br>= neither agree nor disagree<br>= rather disagree<br>= strongly disagree
**SGRolesMenMoneyWomenHouse**	55 (2.2)<br>195 (7.7)<br>585 (23.1)	= strongly agree<br>= rather agree<br>= neither agree nor disagree

The man's job is to earn money; a woman's job is to look after the home and family.	735 (29.0)	= rather disagree
	964 (38.0)	= strongly disagree
	1,251 (49.4)	= strongly agree
**SGRolesGoodRelationshipWorkingMom**	690 (27.2)	= rather agree
A working mother can establish just as warm and secure a relationship with her children as a mother who does not work.	409 (16.1)	= neither agree nor disagree
	157 (6.2)	= rather disagree
	27(1.1)	= strongly disagree
	272 (10.7)	= strongly agree
**SGRolesWomenWorkChildSuffer**	691 (27.3)	= rather agree
A pre-school child is likely to suffer if his or her mother works.	770 (30.4)	= neither agree nor disagree
	506 (20,0)	= rather disagree
	295 (11.6)	= strongly disagree
	88 (3.5)	= strongly agree
**SGRolesWomenWorkFamilySuffer**	389 (15.4)	= rather agree
All in all, family life suffers when the woman is working.	939 (37.1)	= neither agree nor disagree
	699 (27.6)	= rather disagree
	419 (16.5)	= strongly disagree
	327 (12.9)	= strongly agree
**SGRolesHousewifeFulfilling**	440 (17.4)	= rather agree
Being a housewife is just as fulfilling as working for pay.	743 (29.3)	= neither agree nor disagree
	728 (28.7)	= rather disagree
	296 (11.7)	= strongly disagree
	187 (7.4)	= strongly agree
**SGRolesHousehusbandFulfilling**	332 (13.1)	= rather agree
Being a househusband is just as fulfilling as working for pay.	733 (28.9)	= neither agree nor disagree
	884 (34.9)	= rather disagree
	398 (15.7)	= strongly disagree
	232 (9.2)	= strongly agree
**SGRolesSingleParentEqual**	423 (16.7)	= rather agree
One parent can raise a child as well as two parents together.	802 (31.6)	= neither agree nor disagree
	818 (32.3)	= rather disagree
	259 (10.2)	= strongly disagree
	444 (17.5)	= strongly agree
**SGRolesSameSexEqual**	591 (23.3)	= rather agree
A same-sex couple can raise a child as well as a male-female couple.	618 (24.4)	= neither agree nor disagree
	486 (19.2)	= rather disagree
	395 (15.6)	= strongly disagree
**Externalized sex/gender expressions**		
	308 (12.2)	= very masculine
**SGExpressionLooks**	670 (26.4)	= mainly masculine
How would other people generally describe you based on your appearance, clothing style and other visual characteristics?	179 (7.1)	= a little masculine
	227 (9.0)	= just as feminine as masculine
	186 (7.3)	= a little feminine
	690 (27.2)	= mainly feminine
	274 (10.8)	= very feminine
	289 (11.4)	= very masculine
**SGExpressionBehavior**	662 (26.1)	= mainly masculine
How would other people generally describe you based on your behaviors?	218 (8.6)	= a little masculine
	301 (11.9)	= just as feminine as masculine
	171 (6.7)	= a little feminine
	656 (25.9)	= mainly feminine
	237 (9.4)	= very feminine

**SGExpressionSAGE**<br>SGExpressionLooks + SGExpressionBehavior<br>*(seven-point scales ranging from "very feminine" to "very masculine")*<br><br>SAGE-Score values: 1,0 – 7,0<br>low values = high socially assigned gender conformity	642 (25.3)<br>1,292 (51.0)<br>358 (14.1)<br>210 (8.3)<br>30 (1.2)<br>1 (0.0)<br>1 (0.0)<br><br>2.19<br>0.92<br>1.00<br>7.00	= 1,0 – 1,5 *(high socially assigned gender conformity)*<br>= 2,0 – 2,5<br>= 3,0 – 3,5<br>= 4,0 – 4,5<br>= 5,0 – 5,5<br>= 6,0 – 6,5<br>= 7,0 (*low socially assigned gender conformity)*<br><br>= mean<br>= SD<br>= min<br>= max
**<u>Items contributing to explain structural sex/gender relations</u>**		
**Experience of discrimination**		
**DiscriminationSocialPosition**<br>I have the feeling to be disadvantaged because of my position in society.	24 (0.9)<br>125 (4.9)<br>229 (9.0)<br>971 (38.3)<br>1,185 (46.8)	= strongly agree<br>= rather agree<br>= neither agree nor disagree<br>= rather disagree<br>= strongly disagree
**DiscriminationAge**<br>I have the feeling to be disadvantaged because of my age.	26 (1.0)<br>165 (6.5)<br>275 (10.9)<br>936 (36.9)<br>1,132 (44.7)	= strongly agree<br>= rather agree<br>= neither agree nor disagree<br>= rather disagree<br>= strongly disagree
**DiscriminationHeight**<br>I have the feeling to be disadvantaged because of my height.	11 (0.4)<br>41 (1.6)<br>92 (3.6)<br>627 (24.7)<br>1,763 (69.6)	= strongly agree<br>= rather agree<br>= neither agree nor disagree<br>= rather disagree<br>= strongly disagree
**DiscriminationWeight**<br>I have the feeling to be disadvantaged because of my weight.	15 (0.6)<br>46 (1.8)<br>125 (4.9)<br>614 (24.2)<br>1,734 (68.4)	= strongly agree<br>= rather agree<br>= neither agree nor disagree<br>= rather disagree<br>= strongly disagree
**DiscriminationDisability**<br>I have the feeling to be disadvantaged because of my physical impairment.	24 (0.9)<br>58 (2.3)<br>145 (5.7)<br>428 (16.9)<br>1,879 (74.2)	= strongly agree<br>= rather agree<br>= neither agree nor disagree<br>= rather disagree<br>= strongly disagree
**DiscriminationEthnicity**<br>I have the feeling to be disadvantaged because of my ethnic/cultural affiliation.	8 (0.3)<br>10 (0.4)<br>34 (1.3)<br>269 (10.6)<br>2,213 (87.3)	= strongly agree<br>= rather agree<br>= neither agree nor disagree<br>= rather disagree<br>= strongly disagree
**DiscriminationSG**<br>I have the feeling to be disadvantaged because of my sex/gender.	9 (0.4)<br>28 (1.1)<br>88 (3.5)<br>331 (13.1)<br>2,078 (82.0)	= strongly agree<br>= rather agree<br>= neither agree nor disagree<br>= rather disagree<br>= strongly disagree
	8 (0.3)	= strongly agree

**DiscriminationSexualOrientation**	2 (0.1)	= rather agree
I have the feeling to be disadvantaged	18 (0.7)	= neither agree nor disagree
because of my sexual orientation.	153 (6.0)	= rather disagree
	2,353 (92.9)	= strongly disagree
**DiscriminationAskedifParentsBornAbroad**	289 (11.4)	= yes
Have you ever been asked in Germany whether you or your parents were born abroad?	2,245 (88.6)	= no
**Care and household activities**		
**CareActivitiesChildren**	90 (3.6)	= only me
Who is currently taking primary	225 (8.9)	= mainly me
responsibility for the following tasks?	829 (32.7)	= me and other people
("Other people" also includes your	274 (10.8)	= mainly other people
partner)	45 (1.8)	= only other people
	1,071 (42.3)	= not applicable
Care and/or upbringing of your children/grandchildren, driving services for your children/grandchildren		
**CareActivitiesSick**	116 (4.6)	= only me
Who is currently taking primary	199 (7.9)	= mainly me
responsibility for the following tasks?	419 (16.5)	= me and other people
("Other people" also includes your	184 (7.3)	= mainly other people
partner)	50 (2.0)	= only other people
	1,566 (61.8)	= not applicable
Care for disabled, chronically ill or in need of care family members, neighbours or friends		
**CareActivitiesCooking**	673 (26.6)	= only me
Who is currently taking primary	522 (20.6)	= mainly me
responsibility for the following tasks?	626 (24.7)	= me and other people
("Other people" also includes your	483 (19.1)	= mainly other people
partner)	230 (9.1)	= only other people
Cooking		
**CareActivitiesHousework**	558 (22.0)	= only me
Who is currently taking primary	608 (24.0)	= mainly me
responsibility for the following tasks?	833 (32.9)	= me and other people
("Other people" also includes your	478 (18.9)	= mainly other people
partner)	57 (2.2)	= only other people
Housework		
**CareActivitiesGardening**	337 (13.3)	= only me
	490 (19.3)	= mainly me

Who is currently taking primary responsibility for the following tasks? ("Other people" also includes your partner)<br><br>Gardening (during the gardening season)	1,031 (40.7)<br>301 (11.9)<br>68 (2.7)<br>307 (12.1)	= me and other people<br>= mainly other people<br>= only other people<br>= not applicable
**CareActivitiesErrands**<br>Who is currently taking primary responsibility for the following tasks? ("Other people" also includes your partner)<br><br>Errands (shopping, procurement)	501 (19.8)<br>557 (22.0)<br>1,100 (43.4)<br>328 (12.9)<br>48 (1.9)	= only me<br>= mainly me<br>= me and other people<br>= mainly other people<br>= only other people
**CareActivitiesHandicraft**<br>Who is currently taking primary responsibility for the following tasks? ("Other people" also includes your partner)<br><br>Handicraft tasks in the household	478 (18.9)<br>666 (26.3)<br>613 (24.2)<br>594 (23.4)<br>183 (7.2)	= only me<br>= mainly me<br>= me and other people<br>= mainly other people<br>= only other people
**Intersectionality-related social categories**		
**SGRelationsSchoolEducation**<br>School education<br>*(Variable from basic KORA studies S1-S4)*	1,062 (41.9)<br><br>793 (31.3)<br>679 (26.8)	= Degree after German basic secondary school (*Hauptschulabschluss*)<br>= German O-levels (*Mittlere Reife*)<br>= German A-Levels (*Abitur*)
**SGRelationsVocationalEducation**<br>Highest vocational qualification<br>*(Variable from basic KORA studies S1-S4)*	157 (6.2)<br>1,358 (53.6)<br>504 (19.9)<br>22 (0.9)<br>493 (19.5)	= no vocational qualification<br>= vocational school / apprenticeship<br>= technical school / master school<br>= engineering school / polytechnical school<br>= university of applied sciences / university
**SGRelationsEmployment**<br>Are you employed?<br><br>If so: How many hours do you work on average per week (actual working hours)?	1,328 (52.4)<br>13 (0.5)<br>97 (3.8)<br>168 (6.6)<br>176 (6.9)<br>454 (17.9)<br>228 (9.0)<br>70 (2.8)	= no<br>= 1-5 h/week<br>= 6-10 h/week<br>= 11-20 h/week<br>= 21-30 h/week<br>= 31-40 h/week<br>= 41-50 h/week<br>= > 50 h/week
**SGRelationsIncome**<br>How do you assess your financial situation?	295 (11.6)<br>1,645 (64.9)<br>559 (22.1)<br>35 (1.4)	= very good<br>= good<br>= moderate<br>= bad
**SGRelationsFamilySituation**<br>Do you live with a spouse or partner in a shared household?<br>*(Variable from KORA surveys F3, F4, FF4, FIT; the latest available information from each participant was used)*	2,045 (80.7)<br>489 (19.3)	= yes<br>= no

**SGRelationsUrbanisation**	902 (35.6)	= city
Distribution of participants by degree of urbanisation.	1,091 (43.1)	= suburb
	541 (21.3)	= rural

[1]*Original questions were asked in German*

Supplementary Table S2: *Additional 13 sex/gender and intersectionality-related covariates within the INGER KORA FIT sample*

**Covariate Name and question in INGER KORA FIT survey**[1]	**Response categories and distribution in the INGER KORA FIT complete case sample; *N* = 1,687 (100 %)**	
**Items contributing to explain structural sex/gender relations**		
**Intersectionality-related social categories**		
**SGRelationsEmploymentCategories**	755 (44.8)	= a pensioner
Which of the following statements applies to you?	73 (4.3)	= in partial retirement
Are you currently ...	707 (41.9)	= working
	34 (2.0)	= unemployed
	118 (7.0)	= other status *(e.g. housewife/ househusband, in training, other leave of absence)*
**SGRelationsOccupation**	22 (1.3)	= unskilled worker
What position do you have, or did you have in your job?	66 (3.9)	= Civil servant simple activity / semi-skilled worker
+ one of four more detailed follow-up questions depending on the initial response (worker, employee, civil-servant or self-employed)	234 (13.9)	= Civil servant simple service / skilled worker
	98 (5.8)	= Civil servant middle service / skilled worker or foreman
	31 (1.8)	= Master craftsman / foreman
	698 (41.4)	= Civil servants higher service / employee with qualified activity
	100 (5.9)	= Self-employed, max. 1 employee
	386 (22.9)	= Civil servant higher service / employee with highly qualified activity / self-employed academic / self-employed with max. 9 employees
	52 (3.1)	= Employees with extensive managerial responsibilities / self-employed with at least 10 employees
**SGRelationsIncomeEuro**	Continuous	variable (€)
Equivalent income = the household's income divided by the number of consumption units in the household.	1937.00	= mean
	920.34	= SD
	185.00	= min
(To calculate this, participants were asked about their household income and number of households' members and the age of the members)	8000.00	= max
**SGRelationsHouseholdMembers**	314 (18.6)	= 1
How many people live in your household at all times, including yourself?	1,061 (62.9)	= 2
	204 (12.1)	= 3
	78 (4.6)	= 4
	25 (1.5)	= 5
	3 (0.2)	= 6

	0 (0.0)	= 7
	1 (0.1)	= 8
	1 (0.1)	= 9

**SGRelationsDisability**	1,367 (81.0)	= no disability
Do you have a recognized disability?	104 (6.2)	= disability 10 - 30%
If yes, what degree of disability do you	165 (9.8)	= disability 40 - 60%
have?	51 (3.0)	= disability 70-100%

**SGRelationsMobility**	1,239 (73.4)	= I have no problems moving around
Please indicate which of the following	272 (16.1)	= I have little problems moving around
statements best describes your current	137 (8.1)	= I have moderate problems moving around
state of health regarding mobility.	39 (2.3)	= I have big problems moving around / I am unable to move

Life-style and psychological factors

Health Behaviors

**HealthBehaviorAlcohol**	Continuous	variable
Alcohol consumption (g /day)	15.12	= mean
	19.30	= SD
(To calculate this, participants were asked	0.00	= min
about their consumption of different kind of	160.00	= max
alcoholic drinks on weekends or working days)		

**HealthBehaviorSmoking**	191 (11.3)	= regular smoker
Smoking behavior based on three interview	35 (2.1)	= irregular smoker
questions	710 (42.1)	= ex-smoker
a) Do you currently smoke cigarettes?	751 (44.5)	= never-smoker
b) Do you smoke regularly or irregularly (i.e. usually less than one cigarette a day)?		
c) Have you ever smoked cigarettes?		

**HealthBehaviorPhysicalActivity**	655 (38.8)	= regularly > 2h a week
How often do you do sports in	586 (34.7)	= regularly ~ 1h a week
winter/summer?	215 (12.7)	= irregularly ~ 1h a week
	231 (13.7)	= almost no or no physical activity

Psychosocial Factors

**LifeSatisfaction**	Continuous	variable
Overall, how satisfied are you with your life	7.46	= mean
right now?	2.18	= SD
	0.00	= min
on a scale from 0 (totally dissatisfied) – 10	10.00	= max
(completely satisfied)		

PerceivedStress

Based on 10 questions of the German version of the Perceived Stress Scale (see Klein [62]) for exact formulation of questions)<br><br>Scores are obtained by reversing responses to four positively stated items and then summing across all scale items. Higher scores indicated a higher level of perceived stress.	Continuous variable<br>14.31 = mean<br>5.57 = SD<br>0.00 = min<br>34.00 = max
**SelfEfficacy**<br>Self-efficacy scale (ASKU, see Beierlein [63])<br><br>Score is based on three statements on a scale from 1 (strongly disagree) – 5 (strongly agree):<br>a) I can rely on my own abilities in difficult situations.<br>b) I am able to solve most problems on my own.<br>c) I can usually solve even challenging and complex tasks well.<br><br>Score is the average response to these three statements.	Continuous variable<br>4.04 = mean<br>0.57 = SD<br>1.00 = min<br>5.00 = max
**OptimismPessimism**<br>Based on questions of the German version of the Life-Orientation-Test (LOT-R) for dispositional optimism and pessimism [64]<br><br>Scores are obtained by reversing responses to positively stated items and then summing across all scale items. Higher scores indicated a higher level of pessimism.	Continuous variable<br>7.49 = mean<br>3.60 = SD<br>0.00 = min<br>20.00 = max

[1]*Original questions were asked in German*

62. Klein E.M.; Brahler E.; Dreier M.; Reinecke L.; Muller K.W.; Schmutzer G.; Wolfling K.; Beutel M.E. The German version of the Perceived Stress Scale - psychometric characteristics in a representative German community sample. *BMC Psychiatry* **2016**, *16*, 159. doi:10.1186/s12888-016-0875-9
63. Beierlein C.; Kovaleva A.; Kemper C.J.; Rammstedt B. *ASKU - Allgemeine Selbstwirksamkeit Kurzskala [Fragebogen]*. Elektronisches Testarchiv (PSYNDEX Tests-Nr. 9006490). Trier: ZPID.: Leibniz-Zentrum für Psychologische Information und Dokumentation (ZPID); 2012.
64. Scheier M.F.; Carver C.S.; Bridges M.W. Distinguishing optimism from neuroticism (and trait anxiety, self-mastery, and self-esteem): a reevaluation of the Life Orientation Test. *J Pers Soc Psychol* **1994**, *67(6)*, 1063-78. doi:10.1037//0022-3514.67.6.1063

Supplementary Table S3: *Comparison of the whole INGER sample with the complete case sample*

Variable	Response categories	Distribution in the whole INGER sample; *N* = 3,742 (100 %)	Distribution in the complete case sample; *N* = 2,534 (100 %)
**Age in years**	mean =	63.41	62.69
	SD =	9.42	8.87
	min =	43	43
	max =	92	90
**Sex/gender identity**	I don´t want to classify=	14 (0.4)	8 (0.3)
	female =	1,998 (54.3)	1,334 (52.6)
	female and I don´t want to classify =	3 (0.1)	2 (0.1)
	male =	1,650 (44.8)	1,184 (46.7)
	male and I don´t want to classify=	7 (0.2)	2 (0.1)
	male and other identity =	4 (0.1)	2 (0.1)
	other identity =	4 (0.1)	0 (0.0)
	transsexual =	2 (0.1)	2 (0.1)
		60 missings	
**Sex assigned at birth**	female =	2,014 (54.5)	1,339 (52.8)
	male =	1,684 (45.5)	1,195 (47.2)
	divers / intersexual =	0 (0.0)	0 (0.0)
		44 missings	
**School education**	basic secondary school degree =	1,736 (46.4)	1,062 (41.9)
	German O-Levels =	1,085 (29.0)	793 (31.3)
	German A-Levels =	920 (24.6)	679 (26.8)
		1 missing	
**Employment**	Yes =	1,681 (46.0)	1,206 (47.6)
	No =	1,970 (54.0)	1,328 (52.4)
		91 missings	
**Self-rated financial situation**	very good =	394 (10.6)	295 (11.6)
	good =	2,303 (62.2)	1,645 (64.9)
	moderate =	937 (25.3)	559 (22.1)
	bad =	66 (1.8)	35 (1.4)
		42 missings	
**Degree of urbanisation.**	city =	1,139 (34.7)	902 (35.6)
	suburb =	1,392 (42.4)	1,091 (43.1)
	rural =	751 (22.9)	541 (21.3)
		460 missings	
**Self-rated health**	good =	2,728 (73.7)	1,926 (76.0)
	bad =	975 (26.3)	608 (24.0)
		39 missings	
**Access to high quality public green spaces**	high quality green spaces =	1,066 (29.8)	774 (30.5)
	lower quality and no green spaces =	2,513 (70.2)	1,760 (69.5)
		163 missings	
**Greenness in the residential environment**	very green =	2,911 (78.3)	2,008 (79.2)
	less green =	809(21.7)	526 (20.8)
		22 missings	
**Greenness within a 300 m buffer (NDVI)**	mean =	0.47	0.47
	SD =	0.09	0.09
	min =	0.16	0.16
	max =	0.73	0.73

**Greenness within a 1000 m buffer (NDVI)**	mean =	0.50	0.50
	SD =	0.09	0.09
	min =	0.27	0.27
	max =	0.71	0.71
